# The effect of colchicine on coagulation in patients with chronic coronary disease who use vitamin K antagonists

**DOI:** 10.1007/s00228-025-03815-9

**Published:** 2025-03-07

**Authors:** Jeroen P. A. Houwen, Arief Lalmohamed, Jochem Zwaan, Toine C. G. Egberts, Michiel Duyvendak, Aernoud T. L. Fiolet, Arend Mosterd

**Affiliations:** 1 Department of Clinical Pharmacy, University Medical Center Utrecht, Utrecht, The Netherlands; 2 Division of Pharmacoepidemiology and Clinical Pharmacology, Utrecht Institute for Pharmaceutical Science, Faculty of Science, Utrecht University, Utrecht, The Netherlands; 3 Pharmacy de Lindehoeve, Barendrecht, The Netherlands; 4 Antonius Hospital Sneek and Pharmacy d&a Research, Sneek, The Netherlands; 5 Department of Cardiology, University Medical Center Utrecht, Utrecht, The Netherlands; 6 Department of Cardiology, Meander Medical Center, Amersfoort, The Netherlands

**Keywords:** LoDoCo2 Colchicine Drug interaction, VKA: Vitamin-K antagonists, INR: International normalized ratio

## Abstract

**Background:**

Low-dose (0.5 mg/day) colchicine improves cardiovascular outcomes in patients with stable coronary disease. Around 10–15% of these patients simultaneously use anticoagulant therapy, including vitamin-K antagonists (VKAs). In vitro studies and case reports have described a possible interaction between colchicine and VKAs leading to increased INR, but controlled studies are lacking.

**Objective:**

The aim of this study was to investigate if there is a drug-drug interaction between low-dose colchicine and VKAs in patients with chronic coronary disease.

**Methods:**

This study was a sub-analysis of the randomized low-dose colchicine for secondary prevention of cardiovascular disease 2 (LoDoCo2) trial. This placebo-controlled trial investigated efficacy of colchicine 0.5 mg once daily in patients with chronic coronary disease. For the current study, we included a selection of Dutch patients who concomitantly used a VKA. Following a 30 days open-label colchicine run-in phase, patients were randomized to colchicine or placebo. The primary outcome was the intra-patient difference in international normalized ratio (INR) during the first month after *starting* or *stopping* colchicine as compared to the preceding month. Secondary outcomes included changes in VKA daily dosage, assessed in the same pattern and before and after randomization, and time in therapeutic range (TTR), assessed before and after randomization to reflect long-term effects. INR measurements were part of routine clinical care.

**Results:**

In total, 73 patients were included (35 colchicine and 38 in the placebo group). No significant intra-patient change in INR was observed after *starting* colchicine during the open-label run-in phase (mean INR: 2.60 before vs. 2.67 during run-in, difference 0.07, 95% CI − 0.13 to 0.26; *p* = 0.50). Similarly, *stopping* colchicine treatment (i.e., randomization to placebo) did not significantly alter INR levels (mean INR: 2.70 during run-in vs. 2.81 after randomization, difference 0.11, 95% CI − 0.12 to 0.33; *p* = 0.34). The change in mean VKA daily dosage was − 0.01 mg (95% CI − 0.03 to 0.01; *p* = 0.35) when starting colchicine and − 0.01 mg (95% CI − 0.03 to 0.01; *p* = 0.41) when switching to placebo. TTR in patients allocated to active treatment was 65.8% in the year prior to the start of colchicine and 73.4% in the year after randomization to colchicine (change in TTR 7.56%, 95% CI − 0.14 to 15.26%; *p* = 0.05). Mean VKA dosage remained similar (change in VKA dosage of 0.01 mg; 95% CI − 0.11 to 0.13 mg; *p* = 0.84).

**Conclusion:**

No significant changes in INR, VKA dosage, or TTR in patients using VKAs after starting or stopping colchicine were observed. These results suggest that there is no need for additional INR monitoring beyond the standard of care when using low-dose colchicine, though further studies in larger populations would help to confirm this conclusion.

**Supplementary Information:**

The online version contains supplementary material available at 10.1007/s00228-025-03815-9.

## Introduction

Cardiovascular diseases (CVD) are among the leading causes of mortality worldwide [1, 2]. Despite primary and secondary prevention strategies, the morbidity and mortality associated with CVD remains high, underscoring the need for novel therapeutic approaches [3–6]. A systematic review and meta-analysis of randomized trials demonstrated that low-dose colchicine (0.5 mg/day) significantly reduces the risk of major adverse cardiovascular events by 25%, myocardial infarction by 22%, stroke by 46%, and the need for coronary revascularization by 23% [7]. These findings have led to the recommendation of using colchicine in patients with coronary artery disease by European and American guidelines [8, 9].

It is noteworthy that up to 10–15% of these patients concurrently receive anticoagulant medication of which 24% comprises vitamin-K antagonists (VKAs) [7]. Despite the increasing use of direct oral anticoagulants (DOACs), VKAs remain a common choice for anticoagulation, particularly in patients with mechanical heart valves or those with contra-indications for DOACs. Colchicine inhibits CYP2C9 in vitro, the primary enzyme involved in the metabolism of VKAs, which may also occur in vivo [10–12]. A case series report described impaired anticoagulation and bleeding within days of starting colchicine at doses of 1–6 mg in VKA users [13]. However, clinical studies to determine whether there is a true causal relationship are lacking.

This study aims to investigate whether there is a drug-drug interaction between low-dose colchicine (0.5 mg once daily) and VKAs in patients with chronic coronary disease.

## Methods

### Study design and population

This study was a sub-analysis of the “Low-dose colchicine for secondary prevention of cardiovascular disease 2” (LoDoCo2) trial, which was a randomized, double-blind, placebo-controlled study conducted in the Netherlands and Australia from 2014 to 2019. In short, this cardiovascular outcomes trial investigated efficacy of 0.5 mg colchicine daily in patients with chronic coronary syndrome. The design and outcomes of the study have been published elsewhere [3, 14].

**Fig. 1:**
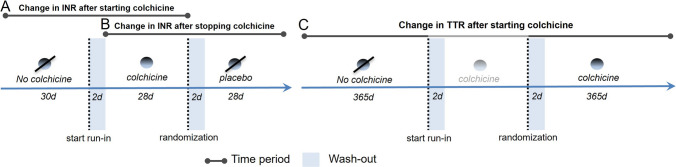
Research timeline overview VKA vitamin-K antagonist, INR international normalized ratio

This sub-analysis included all VKA users from the LoDoCo2 study at three Dutch hospitals: Meander Medical Center (MMC), Northwest Hospital Group (NWG), and Isala Meppel (IM). Patients were treated with acenocoumarol or phenprocoumon, as warfarin is not prescribed in the Netherlands. These large, representative institutions had readily accessible international normalized ratio (INR) measurements. Patients were included if they were treated with VKA and had at least six INR measurements available prior to study participation to ensure continuous use. The INR and VKA dosage data were collected as part of routine care and obtained from the thrombosis services at the participating hospitals. The timeline of the LoDoCo2 and the current study is depicted in Fig. 1. Eligible patients entered an open-label run-in phase for 30 days, in which they received 0.5 mg of colchicine once daily, followed by randomized allocation to either continue with colchicine or switch to placebo. The follow-up period for patients extended from 1 year prior to the study until 1 year after randomization.

### Outcomes

The primary outcome of the sub-study was the intra-patient difference in INR during the first month after *starting* or *stopping* colchicine as compared to the preceding month. Change in INR was measured after the introduction of open-label colchicine during the run-in phase (a pre-post comparison of no colchicine versus colchicine) and change in INR after the introduction of placebo during the randomized phase of the trial (a pre-post comparison of colchicine versus no colchicine). To minimize potential interference from the thrombosis service and accurately assess the potential drug-drug interaction between colchicine and VKAs on INR, an intra-patient comparison approach was used. This was assessed in two phases: (A) after the start of colchicine treatment during the open-label run-in phase (Fig. 1A) and (B) directly after switching to placebo, reflecting the discontinuation of colchicine (Fig. 1B). During these treatment switches, the first INR measurement in the new period was compared to the most recent INR measurement in the preceding time window, allowing a washout of 2 days (i.e., the first 2 days after switching treatment regimen were excluded from the analysis) and minimizing interference from the thrombosis services.

Secondarily, in a similar manner, intra-patient difference in VKA-dosage was compared between the previously defined time windows (Fig. 1).

Finally, to investigate the long-term impact of colchicine on VKA efficacy, time in therapeutic range (TTR) was assessed by comparing each patient’s TTR in the 365 days after randomization with their TTR in the equivalent period before trial participation (Fig. 1C). The TTR was determined using the Rosendaal method, which utilizes linear interpolation to estimate the actual days spent within the therapeutic range [15]. A therapeutic INR was defined as 2.0–3.0 or 2.5–3.5, depending on the indication.

The occurrence of extreme INR values, defined as INR values > 5.0 or < 2.0, was also examined during the 30 days before and after initiating colchicine therapy.

Collected co-variables included age (continuous), sex, VKA type (acenocoumarol and phenprocoumon), VKA indication (atrial fibrillation, mechanical heart valve, pulmonary embolism, venous thromboembolism, thrombus in the heart, stent, cardiomyopathy, and aneurysm), and interacting drugs (i.e., those enhancing or inhibiting the effect of VKAs). The latter were selected from the Medical Pharmaceutical Decision Rules from The Royal Dutch Pharmacists Association KNMP-MFB, Micromedex interaction checker, and the Federation of Dutch Thrombosis Services (FNT). Included drugs are stated in the footnote of Table 1.

**Table 1:** Baseline characteristics of patients

**Demographic data**			
	Age—years	71.4 ± 7.1	69.9 ± 8.5
	Male—n. (%)	28 (80.0)	33 (86.8)
	Female—n. (%)	7 (20.0)	5 (13.2)
**VKA n. (%)**			
	Acenocoumarol	29 (82.9)	30 (79)
	Phenprocoumon	6 (17.1)	8 (21)
**Co-medication n. (%)**			
	Enhanced** effect VKA		
	0	8 (22.9)	9 (23.7)
	1	13 (37.1)	14 (36.8)
	> 1	14 (40.0)	15 (39.5)
	Reduced*** effect VKA		
	0	29 (82.8)	31 (81.6)
	1	6 (17.2)	7 (18.4)
VKA indication n. (%)****			
	Atrial fibrillation	26 (65)	23 (50)
	Mechanical heart valve	1 (2.5)	2 (4.3)
	Pulmonary embolism	0 (0)	2 (4.3)
	Venous thromboembolism	2 (5.0)	1 (2.2)
	Left ventricular thrombus	5 (12.5)	9 (19.6)
	Peripheral stent	2 (5.0)	2 (4.3)
	Cardiomyopathy	0 (0)	3 (6.5)
	Aneurysm	3 (7.5)	2 (4.3)
	Other	1 (2.5)	2 (4.3)
**Target therapeutic INR range n. (%)**			
	Low (2.0–3.0)	30 (85.7)	25 (65.8)
	High (2.5–3.5)	5 (14.3)	13 (34.2)

*VKA* vitami-n-K antagonist, *INR* international normalized ratio

^*^Plus–minus values are means ± SD

^**^Enhancers: see appendix I

^***^Reducers: see appendix I

^****^Patients can have multiple VKA indications

### Analysis

No reliable in vivo estimates of the expected change in INR after introducing colchicine are available in the literature. We aimed for an INR positioned centrally within the therapeutic range—2.5 for low- and 3.0 for high-intensity therapy. Given that a shift of 0.5 in INR would place values outside this target range, we considered this change clinically significant. To detect an INR difference of 0.5, using *α* = 0.05 and *β* = 0.20, a sample size of at least 20 would be required. We deliberately chose this difference in mean INR to capture potential drug-drug interactions effectively.

Continuous variables are presented as means or medians with standard deviations and interquartile ranges for normally and non-normally distributed variables, respectively. Distributions were assessed using the Kolmogorov–Smirnov test, in addition to visual inspection with Q-Q plots and histograms. Categorical variables were presented as counts and percentages.

Intra-patient changes in INR, VKA dosage, and TTR were analyzed using paired *T*-tests with a two-sided significance level of 0.05. The data were stratified by sex, VKA type, age, and the total number of interacting medications each patient was using.

All data handling and statistical analyses were conducted using SAS 9.4.

This study involved a re-analysis of data, with additional data obtained from the Dutch Thrombosis Services. The re-analysis fell under previous medical ethics approval and was conducted in compliance with existing approvals ACTRN12614000093684.

## Results

### Patient selection

**Fig. 2:**
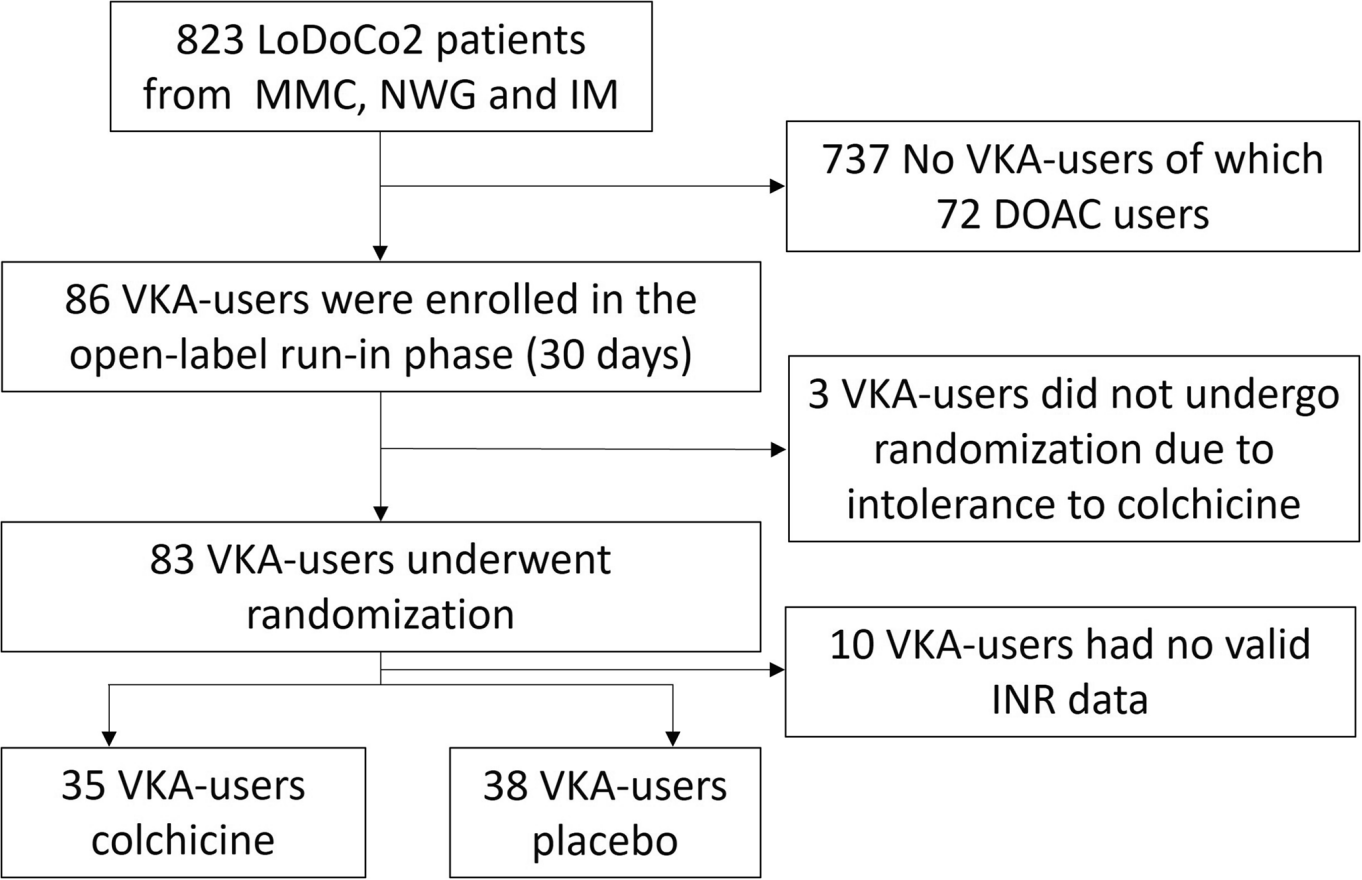
Patient selection flowchart LoDoCo2: Low-Dose Colchicine 2, MMC: Meander Medical Center (22 patients), NWG: Northwest Hospital Group (38 patients), IM: Isala Meppel (26 patients), VKA: Vitamin-K Antagonist, DOAC: Direct Oral Anticoagulant, INR: International Normalized Ratio

Figure 2 presents a flow-chart of patient selection. Three participating centers enrolled a total of 73 patients who were concomitantly taking a VKA and had available INR data. Of these 73 patients, 35 were randomized to colchicine and 38 to placebo (Table 1). The mean age of patients was 70.6 years. There were 12 (16%) female patients. The majority of patients used acenocoumarol (81%). Atrial fibrillation was the most common indication for VKA use. The use of potentially interacting comedications was similar between the two groups.

### Changes in INR after starting colchicine

Following the *start* of colchicine in the open-label run-in phase, no significant intra-patient change in INR was observed among the 57 patients with available INR measurements during this phase. The mean last measured INR prior to the open-label run-in phase was 2.60, as compared to 2.67 during the open-label run-in phase (INR difference 0.07, 95% confidence interval [CI] − 0.13 to 0.26; *p* = 0.50), as shown in Table 2. The mean VKA dosage was 2.53 mg in the period before the open-label run-in phase, as compared to 2.52 mg during the open-label run-in phase (VKA dosage difference − 0.01 mg, 95% CI − 0.03 to 0.01; *p* = 0.35). Furthermore, no differences in INR or VKA dosage were found when stratifying by sex, type of VKA, age, and interacting medications. Additionally, no significant differences were observed in extreme INR values: one patient had an INR greater than 5.0, compared to two patients in the 30 days before starting colchicine. Similarly, 11 patients had INR values below 2.0 after starting colchicine, compared to 14 patients in the 30 days before starting colchicine.

**Table 2:** INR after starting and stopping colchicine

**Starting with colchicine**	
Change in INR (*n* = 57)	
INR before starting colchicine	2.60
INR immediately after starting colchicine	2.67
Change in INR (95% CI)	0.07 (− 0.13 to 0.26; *p* = 0.50)
Change in VKA dosage (*n* = 47)	
Dosage before starting colchicine (mg)	2.53
Dosage immediately after starting colchicine (mg)	2.52
Dosage change (95% CI)	− 0.01 (− 0.03 to 0.01; *p* = 0.35)
**Stopping colchicine**	
Change in INR (*n* = 32)	
INR with colchicine	2.70
INR immediately after stopping colchicine	2.81
Change (95% CI)	0.11 (− 0.12 to 0.33; *p* = 0.34)
Change in VKA-dosage (*n* = 24)	
Dosage with colchicine (mg)	2.30
Dosage after stopping colchicine (mg)	2.29
Dosage change (95% CI)	− 0.01 (− 0.03 to 0.01; *p* = 0.41)

Data are presented as means. Mean differences are reported with 95% confidence intervals (CI)

*INR* international normalized ratio, *CI* confidence interval, *VKA* vitamin-K antagonist

### Changes in INR after stopping colchicine

*Stopping* colchicine treatment (i.e., randomization to placebo) did not result in a significant change in INR. The mean last INR was 2.70 during the open-label colchicine run-in phase as compared to a mean INR of 2.81 directly after randomization to placebo (INR difference 0.11, 95% CI − 0.12 to 0.33; *p* = 0.34). Similarly, the mean VKA dosage did not change significantly after stopping colchicine: the mean VKA dosage was 2.30 mg during the open-label colchicine run-in phase as compared to a mean VKA dosage of 2.29 mg directly after randomization to placebo (VKA dosage difference − 0.01 mg, 95% CI − 0.03 to 0.01; *p* = 0.41). No differences in INR and VKA dosage were found when stratifying by sex, type of VKA, age, and interacting medication.

### Effect on time in therapeutic range (TTR) 365 days after randomization

TTR was 65.8% in the year prior to randomization compared to 73.4% during the first year of treatment with colchicine (difference 7.56%, 95% CI − 0.14 to 15.26%; *p* = 0.05), as shown in Table 3. A similar numerical but not statistical significant increase was observed in the placebo group, with a TTR of 66.5% in the year prior to randomization compared to 72.5% during the first year of study (difference 5.98%, 95% CI − 0.70 to 12.66%; *p* = 0.08). VKA dosage did not change significantly, with a change of − 0.01 mg (95% CI − 0.11 to 0.13 mg; *p* = 0.84) in the colchicine group and 0.06 mg (95% CI − 0.04 to 0.15 mg; *p* = 0.26) in the placebo group.

**Table 3:** One-year TTR before and after randomization

**Change in TTR (*n*** **= 35)**	
TTR before starting colchicine (%)	65.8
TTR after randomization to colchicine (%)	73.4
TTR change (95% CI)	7.56 (− 0.14 to 15.26; *p* = 0.05)
**Change in VKA-dosage (*n* = 29)**	
Dosage before starting colchicine (mg)	2.51
Dosage after randomization to colchicine (mg)	2.50
Dosage change (95% CI)	− 0.01 (− 0.11 to 0.13; *p* = 0.84)

*TTR* time in therapeutic range, *CI* confidence interval, *VKA* vitamin-K antagonist

Data are presented as means. Mean differences are reported with 95% confidence intervals (CI)

### Occurrence of extreme INR values

In the 30 days before the start of the open-label run-in, two patients had INR values greater than 5.0, compared to one patient after starting colchicine. Similarly, 14 patients had INR values below 2.0 before the start, compared to 11 patients after starting colchicine. Although the sample size limits definitive conclusions, no obvious differences were observed in the occurrence of extreme INR values after starting or stopping colchicine therapy.

## Discussion

In this sub-analysis of the LoDoCo2 trial, we investigated the potential interaction between low-dose colchicine and VKAs in patients with chronic coronary disease. We found no significant changes in INR, VKA dosage, or TTR in patients using VKAs after starting or stopping colchicine. These results suggest that additional INR monitoring beyond the standard of care may not be necessary when using low-dose colchicine. Up to 25% of patients with coronary artery disease use an anticoagulant. The annual decrease in VKA use is stabilizing, indicating it will likely remain a part of clinical practice for the foreseeable future [16, 17].

Contrary to our findings, earlier in vitro studies suggested changes in INR with the use of colchicine [18]. In this study, colchicine concentrations of 1 µmol/L were used, corresponding with 399 µg/L, which was approximately 200 times higher than what would be expected from pharmacokinetic studies after oral ingestion of 0.6 mg colchicine reaching between 1 and 3 µg/L [19, 20]. Additionally, case reports that described changes in effect of anticoagulation treatment all used higher daily doses of colchicine (1–6 mg/day) [13]. These findings and those from our study suggest a dose responsive effect with clinical relevant changes only appearing at higher concentrations than those reached with a dose of 0.5 mg once daily and physiological elimination of the drug.

Our study was powered to detect moderate INR differences. With 57 paired samples, the post-hoc power analysis showed 99.9% power for a 0.5 INR difference and 80% power for a 0.28 difference. Given that normal INR fluctuations are around ± 0.25 in stable patients, missing smaller changes below 0.28 would not be clinically concerning, as they likely reflect normal variability rather than a true drug-drug interaction [21].

An INR above the target range increases the risk of bleeding. In our study, INR monitoring followed standard practice, with intervals typically between 1 and 6 weeks [21]. Extreme values were defined as INR > 5.0 or < 2.0. We did not observe a difference between the groups in terms of episodes of extreme INR measurements. Typically, INR control intervals range from 1 to 6 weeks [21].

This study has several limitations. First, the sample size was relatively small, limiting the ability to detect subtle effects in a broader population. Although our study was sufficiently powered for INR changes of > 0.28, larger-scale studies would further substantiate our findings.

Second, real-world adherence and INR variability may differ from our study setting, as patients in clinical trials often receive more structured monitoring and support, potentially leading to better adherence to VKA therapy compared to routine clinical practice. This may have contributed to the observed stability in INR and TTR. To minimize this potential clinical trial effect, we specifically used the last INR measurement before starting colchicine and the first INR measurement after initiation to assess intra-patient differences to reflect any direct pharmacokinetic effects. Additionally, the increase in TTR during the study period, although not statistically significant, could suggest a Hawthorne effect, where patient behavior improves due to participation in the clinical study. This was observed in both the colchicine and placebo group.

Third, the absence of genotyping for CYP enzymes, particularly given that polymorphisms could influence the extent of the interaction. For instance, in individuals who are poor CYP2C9 metabolizers (PM), the potential interaction may be more pronounced, potentially skewing the results. However, this concern is likely mitigated in our study due to the randomization process, which should ensure an approximately equal distribution of PM CYP2C9 patients across both the treatment and placebo groups.

Finally, this study did not include warfarin users, as warfarin is not prescribed in the Netherlands. Given the similar CYP2C9 metabolism of warfarin, acenocoumarol, and phenprocoumon, our findings are likely generalizable [22]. However, given the potential pharmacokinetic differences between VKAs, further research is necessary to confirm their applicability to warfarin-treated populations.

## Conclusion

In patients with chronic coronary diseases treated with a VKA, starting, using, or stopping low-dose colchicine at a dosage of once daily 0.5 mg does not affect INR, VKA dosage, and TTR. These results are reassuring and support that additional INR measurements—beyond the regular care provided by thrombosis services—are not necessary in patients using a VKA, though further studies in larger populations would help to confirm this conclusion.

## Supplementary Information

Below is the link to the electronic supplementary material.

Supplementary file1 (PDF 89.9 KB)

## Data Availability

No datasets were generated or analysed during the current study.
