# Supplementary material for: The effect of colchicine on coagulation in patients with chronic coronary disease who use vitamin K antagonists
Source: Eur J Clin Pharmacol. 2025 Mar 7;81(5):719–25. doi: 10.1007/s00228-025-03815-9 (PMC12003603; doi:10.1007/s00228-025-03815-9)
Supplement: Supplementary file 1 — Supplementary file1 (PDF 89.9 KB) [file 228_2025_3815_MOESM1_ESM.pdf]

**Enhancers:

Abiraterone, acalabrutinib, allopurinol, amikacin, amiodarone, amoxicillin, atorvastatin, azithromycin, beclometasone, benzbromarone, benzylpenicillin, betamethasone, bezafibrate, bicalutamide, capecitabine, carbamazepine, cefaclor, cefalexin, cefalotin, cefazolin, cefotaxime, cefpodoxime, cefradine, ceftazidime, ceftibuten, ceftriaxone, cefuroxime, ciprofibrate, ciprofloxacin, clarithromycin, clindamycin, clofazimine, colistin, co-trimoxazole, cortisone, dalbavancin, danazol, dapsone, darolutamide, demeclocycline, dexamethasone, disopyramide, disulfiram, doripenem, doxycycline, duloxetine, eravacycline, ertapenem, esomeprazole, ethambutol, fenofibrate, fidaxomicin, fluconazole, fluorouracil, fluvastatin, fosfomycin, fusidic acid, gemfibrozil, gentamicin, hydrocortisone, ibrutinib, imipenem, isoniazid, itraconazole, ketoconazole, leflunomide, levothyroxine, levofloxacin, liothyronine, linezolid, metformin, methenamine, miconazole, minocycline, metronidazole, moxifloxacin, neomycin, netilmicin, nilutamide, nitrofurantoin, norfloxacin, noscapine, ofloxacin, omeprazole, paromomycin, phenethicillin, phenoxymethylpenicillin, phenytoin, pipemidic acid, piperacillin, pivmecillinam, piroxicam, prasterone, pravastatin, prednisolone, prednisone, propafenone, pyrazinamide, quinidine, rosuvastatin, roxithromycin, simvastatin, sitagliptin, sulfadiazine, sulfamethoxazole, tamoxifen, tedizolid, teicoplanin, teriflunomide, testosterone, tetracycline, tigecycline, tobramycin, trazodone, trimethoprim, valproate, vancomycin, venlafaxine, voriconazole

***Reducers:

Apalutamide, aprepitant, azathioprine bosentan carbamazepine colestyramine enzalutamide flucloxacillin fosaprepitant, hypericum, mercaptopurine, metformin, nevirapine, phenobarbital, phenytoin, primidone, rifabutin, rifampicin, rifaximin, ritonavir thiamazole
